# Intrusive effects of semantic information on visual selective attention

**DOI:** 10.3758/s13414-016-1156-x

**Published:** 2016-07-05

**Authors:** George L. Malcolm, Michelle Rattinger, Sarah Shomstein

**Affiliations:** 1 School of Psychology, University of East Anglia, Norwich, UK; 2 Department of Psychology, George Washington University, Washington, DC USA

**Keywords:** Visual attention, Semantic information, Scene processing

## Abstract

Every object is represented by semantic information in extension to its low-level properties. It is well documented that such information biases attention when it is necessary for an ongoing task. However, whether semantic relationships influence attentional selection when they are irrelevant to the ongoing task remains an open question. The ubiquitous nature of semantic information suggests that it could bias attention even when these properties are irrelevant. In the present study, three objects appeared on screen, two of which were semantically related. After a varying time interval, a target or distractor appeared on top of each object. The objects’ semantic relationships never predicted the target location. Despite this, a semantic bias on attentional allocation was observed, with an initial, transient bias to semantically related objects. Further experiments demonstrated that this effect was contingent on the objects being attended: if an object never contained the target, it no longer exerted a semantic influence. In a final set of experiments, we demonstrated that the semantic bias is robust and appears even in the presence of more predictive cues (spatial probability). These results suggest that as long as an object is attended, its semantic properties bias attention, even if it is irrelevant to an ongoing task and if more predictive factors are available.

Our environment contains more visual information than we can process in a given moment due to capacity limitations within the retina and the cerebral cortex. The visual system has evolved to deal with this limitation by selecting, or attending to, a subset of the available stimulation considered to be important (Castelhano, Mack, & Henderson, 2009; Yarbus, 1967). Selective attention devotes limited processing capacity to task-relevant or salient information, facilitating the viewer’s current goals. A fundamental objective in the study of human behavior, therefore, is to understand the properties that constrain attentional selection.

Decades of research has demonstrated that the attentional system takes advantage of a range of properties within the environment for the purposes of selection. For instance, low-level physical factors such as spatial location can bias attention (Posner, Snyder, & Davidson, 1980), as can object boundaries (Egly, Driver, & Rafal, 1994; Malcolm & Shomstein, 2015; Pajak & Nuthmann, 2013; Shomstein & Behrmann, 2008) and features (Treisman & Gelade, 1980; Wolfe, 1994). In addition, high-level properties in our surroundings, such as meaning, can also bias attentional selection. For example, a scene’s gist biases attention when looking for a target (Eckstein, Drescher, & Shimozaki, 2006; Malcolm & Henderson, 2010; Neider & Zelinsky, 2006; Spotorno, Malcolm, & Tatler, 2014, 2015; but see Castelhano & Heaven, 2011), even with very short presentation durations (Castelhano & Henderson, 2007; Hillstrom, Scholey, Liversedge, & Benson, 2012; Võ & Henderson, 2010). Similarly, an object’s high-level meaning can bias attentional allocation to semantically related distractors. For instance, you are more likely to fixate a ceramic mug when looking for a coffee machine than if you had been looking for a notebook (Belke, Humphreys, Watson, Meyer, & Telling, 2008; de Groot, Huettig, & Olivers, 2016; Hwang, Wang, & Pomplun, 2011; Mack & Eckstein, 2011; Moores, Laiti, & Chelazzi, 2003).

An important aspect of the previous studies showing that object semantics influence attentional allocation is that these experiments tended to use real-world objects as the search targets and distractors. As such, the high-level meaning of the target was always task-relevant, making semantics central to the successful completion of the task. There is also direct evidence that task-relevant objects readily elicit context-specific activation (Auckland, Cave, & Donnelly, 2007; Bar & Aminoff, 2003; Bar, Aminoff, & Schacter, 2008; Çukur, Nishimoto, Huth, & Gallant, 2013; Davenport & Potter, 2004) and influence early stages of vision, such as parallel processing (Belke et al., 2008) and figure–ground separation (Cacciamani, Mojica, Sanguinetti, & Peterson, 2014). Such observations, pointing to regular extraction of high-level properties and their fast influence on vision, raises the question of whether an object’s semantic properties influence attention even when they are irrelevant to an ongoing task. Importantly, whether semantic information influences attentional allocation independent of its relevance to an ongoing task has not been investigated, and thus remains an open question. Would merely attending to a real-world object—when its meaning is irrelevant—similarly activate semantic knowledge and bias attention to a semantically related object in the scene?

Here, we hypothesized that semantic relationships among objects serve to constrain attentional selection, independent of their task relevance. To foreshadow, Experiments 1–5 showed that the semantic properties of viewed objects bias attention even when they are task-irrelevant. We also hypothesized that if task-irrelevant semantic biasing is robust, it must occur regardless of what other predictive information may be present in the scene (Exps. 6–8). To test the semantic influence on attentional allocation, participants were presented with displays consisting of three real-world objects: one central and, after a variable delay, two peripheral objects. Critically, one of the two peripheral objects was semantically related (SR) to the central object, while the other was nonrelated (NR). A target and two distractors were then presented, superimposed on the objects. Importantly, the target occurred equally on the SR and NR objects, making their respective semantic relationships irrelevant to predicting the target location. If semantic information biases attentional allocation independent of task relevance, then the time to locate the target should be affected by the objects’ semantic relations to the central object. To test the second prediction, that semantic information biases attentional allocation even when alternative predictive factors are available, we introduced an independent spatial probability bias (Exps. 6–8).

Since task-irrelevant semantic biasing of attentional allocation has not been studied extensively, our aim was threefold: (i) to demonstrate that nonpredictive semantic information influences attentional allocation, and that it does so robustly; (ii) to map out the temporal profile of semantic influence by varying the times that objects were visible on the screen prior to target onset; and (iii) to probe whether semantic influence is robust and automatic.

## Experiment 1

The aim of the first experiment was to demonstrate that the task-irrelevant semantic relationships shared between two objects bias attentional selection. A central object appeared on the screen and remained there for 1.5 s, after which two objects (one semantically related to the central object) appeared, arranged equidistantly from the central reference object. Targets appeared on the central object on 50 % of the trials, with the remaining targets distributed equally between the two remaining objects. The logic was as follows: Once the central object was presented, it was attentionally selected and prioritized, given that half of the targets appeared in that spatial location. Following selection of the central object, if semantics guides attentional selection, targets that appeared on the object that was semantically related to the central object should be processed faster and more accurately.

Additionally, the temporal profile of semantic influence was examined by focusing on two time intervals. These intervals were chosen by following the results observed by de Groot and colleagues (2016), who demonstrated that when semantics was relevant to the task, its influence on attention was observed around the 300-400 ms mark. Given that in the de Groot et al. (2016) study, semantics was relevant to the task, and in our experiment semantics was irrelevant, we expected that any semantic contribution would be delayed. Therefore, the two intervals probed in this experiment were restricted to 750 and 1,250 ms.

## Method

### Participants

Twenty-three participants took part in Experiment 1 (13 female, mean age 20.0 years). All were from George Washington University (GWU), gave informed consent, and were naïve to the purpose of the experiment. The experimental procedures were approved by the GWU Institutional Review Board.

### Stimuli and design

**Fig. 1:**
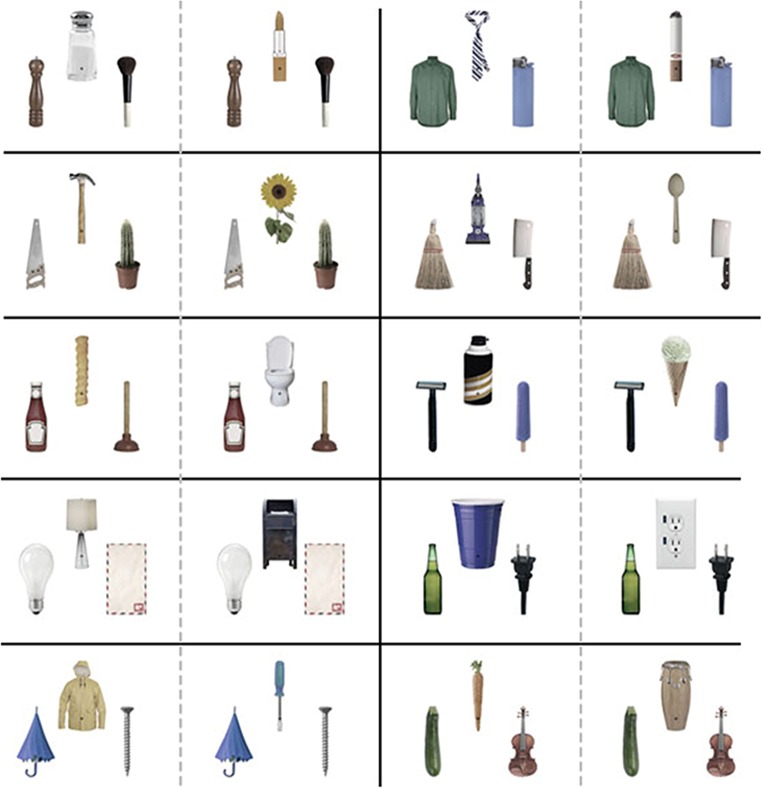
Complete set of stimuli used. We created ten base pairs of peripheral objects, seen in the ten boxes outlined by the solid black lines. Peripheral objects were shown on each side of the screen equally. Each base pair also had two central objects associated with it, one of which appeared during each trial. The central objects were selected to be semantically relevant to only one of the two peripheral objects. For example, in the top-left box, the salt shaker is related to the pepper grinder, whereas the lipstick is related to the make-up brush

Forty upright objects (obtained from Google Images or in-house photos) were selected and scaled to 2.7° in height, with widths varying from 0.4° to 2.1° (Fig. 1). The objects were partially desaturated in Photoshop (Photoshop CS; Adobe, San Jose, California), to reduce potential low-level attentional biasing and make the red target and distractors more visible. Objects were arranged in triads: one centrally located object with a fixation cross at the bottom of it, and two peripheral objects centered 1.8° to either side of the fixation cross. The objects were organized into ten groups, defined by a peripheral object pair (e.g., a make-up brush and pepper grinder) that occurred equally on either side of the screen, and two central objects (e.g., lipstick and a saltshaker), only one of which appeared in a given trial. The central object was semantically related to one of the two peripheral objects (e.g., the lipstick was related to the make-up brush, and the saltshaker to the pepper grinder; see Fig. 1, top left). A target (a red T or L) and two distractors (red T/L hybrids) appeared on every trial at the tops of the objects, in any cardinal orientation. All target/distractor items were equidistant from the fixation cross. Targets appeared 50 % of the time on the central object and 25 % of the time on either of the peripheral objects.

### Procedure

**Fig. 2:**
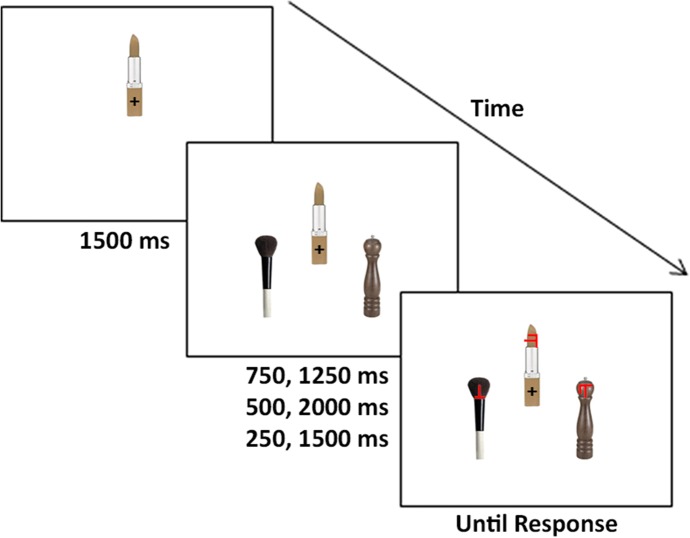
A single trial consisted of a central object appearing on the screen for 1,500 ms. Participants were asked to fixate the central fixation cross throughout the trial. Two peripheral objects then appeared for either 250 or 1,500 ms, 500 or 2,000 ms, or 750 or 1,250 ms depending on the experiment. The target and distractor items then appeared on the objects, and participants had to respond as quickly and accurately as possible if the target was a T or an L

The experimental paradigm was controlled by E-Prime (Sharpsburg, PA). A central object was presented in isolation for 1,500 ms, followed by the onset of a peripheral object pair. The three objects then remained on the screen for either 750 or 1,250 ms, after which time the target/distractor items were presented. Participants were instructed to keep their eyes fixated on the central fixation cross and perform a T/L discrimination task (Fig. 2). Trials were separated by a 500 ms intertrial interval, during which, if the responses were incorrect, the word “incorrect” was flashed in the center.

Participants were given 15 practice trials, followed by four blocks of 160 trials. In each block the stimuli appeared in random order, and the object exposure timings were selected randomly from the two options, with a reset after each two trials. At the end of each block, participants were informed of their current accuracy and mean response time (RT). The experiment lasted approximately 45–50 min.

## Results and discussion

Participants’ median RTs and accuracies were collected. Two participants were removed for having overall median RTs that exceeded 2.5 standard deviations from the grand mean. See Table 1 for RTs.

**Table 1:** Response times (in milliseconds) and standard errors of the means (*SEM*s)

	Condition	250	500	750	1,250	1,500	2,000
**Exps. 1–3**	Valid	726 (26)	726 (24)	747 (25)	731 (25)	719 (24)	725 (25)
	SR	835 (33)	840 (30)	872 (25)	858 (25)	839 (29)	861 (36)
	NR	842 (32)	848 (29)	899 (30)	866 (26)	826 (28)	835 (29)
**Exp. 4**	Valid			722 (22)		749 (31)	
	SR			733 (25)		783 (33)	
	NR			740 (26)		788 (32)	
**Exp. 5**	SR			677 (17)			
	NR			680 (17)			
**Exps. 6–8**	*Valid*	680 (21)	699 (16)	703 (26)	690 (27)	696 (24)	706 (17)
	SR, High %	794 (25)	774 (24)	804 (26)	786 (26)	788 (23)	786 (22)
	NR, High %	775 (24)	779 (22)	809 (26)	797 (26)	785 (23)	771 (24)
	SR, Low %	852 (25)	840 (29)	882 (34)	864 (35)	852 (32)	859 (29)
	NR, Low %	843 (26)	824 (29)	862 (35)	893 (39)	878 (31)	839 (28)

SR, semantically related peripheral object; NR, nonrelated peripheral object; High/low prob., high-/low-probability peripheral location

### Probability manipulation check

Targets occurred on the central object on 50 % of the trials, making it behaviorally relevant. To verify that participants were prioritizing attention to the central object at each exposure duration, the median RTs and accuracy were analyzed in a 2 × 2 repeated measures analysis of variance (ANOVA), with target location (central object and the average of the SR and NR peripheral objects) and exposure duration (750 and 1,250 ms) as factors.

We observed a main effect of target location on RTs [*F*(1, 20) = 74.15, *p* < .001, *η*_p_^2^ = .788], with faster responses for targets on the central than on the peripheral objects (739 and 874 ms, respectively). We also found a main effect of exposure duration [*F*(1, 20) = 5.94, *p* = .024, *η*_p_^2^ = .229], with shorter times in the 1,250 ms condition (816 and 796 ms for the 750 ms and 1,250 ms object exposures, respectively), but no interaction between the factors (*F* < 1). Accuracy similarly showed a main effect of target location [*F*(1, 20) = 10.53, *p* = .004, *η*_p_^2^ = .345], with more accurate responses for identifying targets that appeared on the central object (96.2 % and 93.7 %). There was no main effect of exposure duration on accuracy [*F*(1, 20) = 1.69, *p* = .209, *η*_p_^2^ = .078; 95.2 % and 94.7 %, respectively, for the 750 ms and 1,250 ms conditions], nor an interaction between target location and exposure duration (*F* < 1). The combined results indicate that the intended central object probability bias was effective across both exposure durations.

### Semantic bias

The probability analysis above suggests that attention was initially biased to the central object, and then to the peripheral objects. If the semantic relationship between the central object and the peripheral objects biased attention, then we should observe a difference between the SR and NR RTs.

A two-way, repeated measures ANOVA was run with semantic relation (SR and NR) and exposure duration (750 and 1,250 ms) as within-subjects factors. A main effect emerged of semantic relation on RTs [*F*(1, 20) = 5.26, *p* = .033, *η*_p_^2^ = .208], with targets responded to faster on SR objects (865 ms) than on NR objects (882 ms), and a strong trend for a main effect of exposure duration, with participants finding the target faster after 1,250 ms [*F*(1, 20) = 4.14, *p* = .055, *η*_p_^2^ = .171; 886 and 862 ms for the 750 ms and 1,250 ms durations, respectively], but no interaction between the two [*F*(1, 20) = 2.31, *p* = .144, *η*_p_^2^ = .103]. Accuracy did not show any main effects of semantic relation (*F* < 1) or exposure duration [*F*(1, 20) = 1.52, *p* = .232, *η*_p_^2^ = .071], nor any interaction (*F* < 1).

**Fig. 3:**
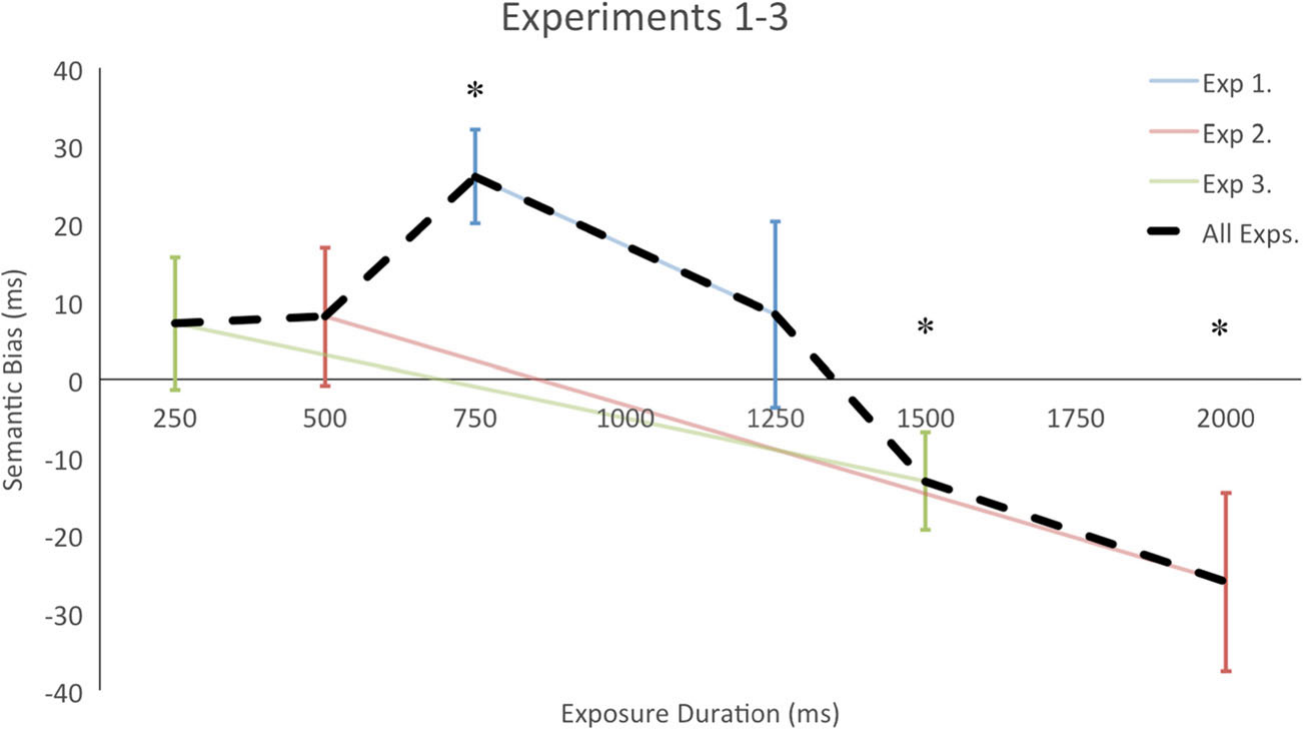
Experiments 1–3: Semantic biases (differences in response times between the nonrelated [NR] and semantically related [SR] conditions) over the six exposure durations. Positive numbers represent a bias toward the SR object; negative numbers, a bias toward the NR object. Error bars represent one standard error

As predicted, we found an attentional bias toward the SR object, despite the fact that the semantic relationship between the peripheral objects and the central object was not predictive of target location (Fig. 3). The results thus suggest that semantic information is incorporated into the attentional allocation processes even when it is irrelevant to the ongoing task, biasing attention to semantically related items.

Although this experiment definitively demonstrated semantic biasing of attention at 750 and 1,250 ms following the presentation of semantically related and nonrelated objects, it left open the questions of whether semantic bias occurs prior to 750 ms or persists following 1,250 ms. Indeed, when semantic information is task-relevant, as in de Groot et al. (2016), semantically related distractors are first fixated around the 300–400 ms mark, suggesting that an earlier semantic bias may not have been probed in Experiment 1. Additionally, we cannot determine whether the SR bias we found is transient or fixed: That is, do participants attend to the SR object for a finite epoch, or do they linger on it until target onset?

## Experiment 2

To test both earlier and later time points of the semantic bias, in Experiment 2 we probed two new exposure durations: 500 and 2,000 ms. This manipulation would examine whether the semantic effect found at 750 and 1,250 ms is stable or transient. If the semantic effect is stable, we should still see a bias toward the SR object at 2,000 ms; if the effect is transient, the effect should disappear or reverse.

## Method

### Participants

Thirty-two participants took part in Experiment 2 (26 female, mean age 19.8 years). All were from GWU, gave informed consent, and were naïve to the purpose of the experiment. All experimental procedures were approved by the GWU Institutional Review Board.

### Stimuli, design, and procedure

The stimuli and design were the same as in Experiment 1, with the only exception being that the exposure durations were now 500 and 2,000 ms.

## Results and discussion

Median RTs and accuracies were collected. One participant was removed for having an overall median RT 2.5 standard deviations longer than the grand mean, and one was removed for having an overall accuracy below 80 %. See Table 1 for RTs.

### Probability manipulation check

Our analyses revealed a main effect of target location on RTs [*F*(1, 29) = 82.12, *p* < .001, *η*_p_^2^ = .739], with faster responses to targets on the central object than to those on the peripheral objects (726 and 846 ms, respectively). However, we found no main effect of exposure duration, and no interaction between the factors (*F*s < 1). Accuracy similarly showed a main effect for target location [*F*(1, 29) = 14.59, *p* = *.*001, *η*_p_^2^ = .335], with more accurate responses for identifying targets appearing on the central object (95.6 %, vs. 93.2 % for the peripheral objects), but no main effect of exposure duration, nor an interaction (*F*s < 1). Just as in Experiment 1, the results indicated that the intended central object probability bias was effective, with faster and more accurate responses to targets on the central as compared to peripheral objects.

### Semantic bias

A two-way, repeated measures ANOVA was run with semantic relation (SR and NR) and exposure duration (500 and 2,000 ms) as within-subjects factors. There was no main effect of semantic relation on RTs [*F*(1, 29) = 1.91, *p* = .177, *η*_p_^2^ = .062], and no effect of exposure duration (*F* < 1). However, we did observe an interaction [*F*(1, 20) = 4.65, *p* = .040, *η*_p_^2^ = .138]. Follow-up paired *t* tests showed no semantic bias toward the SR object at 500 ms (*t* < 1; SR = 840 and NR = 848 ms), but a significant difference toward the NR object at 2,000 ms [*t*(29) = 2.28, *p* = .030, d_z_ = .42; SR = 861 and NR = 835 ms]. Accuracy showed no main effect of semantic relationship or exposure duration (*F*s < 1), but there was a marginal trend toward an interaction [*F*(1, 29) = 3.53, *p* = .070, *η*_p_^2^ = .108], driven by higher accuracy for the SR object at 500 ms and for the NR object at 2,000 ms, though neither of these were significantly different.

In summary, we did not find a bias toward the SR object at 500 ms, which suggests that although task-irrelevant semantic information can bias attention (see Exp. 1), it needs a longer interval of time than when the information is relevant to the task (cf. de Groot et al., 2016). Interestingly, we found that at a later time point (2,000 ms) there was in fact a bias toward the NR object, suggesting transience of the initial SR object bias. In combination with Experiment 1, the results thus suggest that a slow-acting utilization of semantic information to bias attention toward SR objects disappears, and even inverts, in later epochs.

## Experiment 3

Although Experiment 2 demonstrated that a semantic bias is not present at 500 ms, but only emerges at 750 ms (Exp. 1), the bias could have a cyclical nature. In other words, semantic bias could emerge early, and then cycle through epochs of influence. In this experiment, two additional object exposure durations were used, one apiece probing semantic influence at 250 ms (the earliest object exposure) and 1,500 ms.

## Method

### Participants

Twenty-two participants took part in Experiment 3 (13 female, mean age 19.2 years). All were from GWU, gave informed consent, and were naïve to the purpose of the experiment. The experimental procedures were approved by the GWU Institutional Review Board.

### Stimuli, design, and procedure

The stimuli and design were the same as in Experiments 1 and 2, with the only exception being that the exposure durations were now 250 and 1,500 ms.

## Results and discussion

Median RTs and accuracies were collected. One participant was removed for having an overall median RT 2.5 standard deviations longer than the grand mean, and one was removed for having an overall accuracy below 80 %. See Table 1 for RTs.

### Probability manipulation check

We found a main effect of target location on RTs [*F*(1, 19) = 49.00, *p* < .001, *η*_p_^2^ = .721], with faster responses to targets on the central object than to those on the peripheral objects (722 and 836 ms, respectively). However, no main effect of exposure duration emerged, and no interaction (*F*s < 1). There was a main effect of target location on accuracies [*F*(1, 19) = 6.63, *p* = .019, *η*_p_^2^ = .259], with more accurate responses for identifying targets appearing on the central object (95.6 %, vs. 94.4 % on peripheral objects), but no main effect of exposure duration (*F* < 1). However, an interaction did emerge between the effects of target location and exposure duration on accuracy [*F*(1, 19) = 6.87, *p* = .017, *η*_p_^2^ = .265]. Paired *t* tests showed that this was due to responses being significantly more accurate to targets on the central object than to those on peripheral objects at 250 ms [*t*(19) = 3.26, *p* = .004, d_z_ = .73; 95.8 % vs. 93.9 %], and responses being nonsignificantly higher for the central object at 1,500 ms [*t*(19) = 1.03, *p* = .315, d_z_ = .23; 95.4 % vs. 94.9 %]. These results show, as in Experiments 1 and 2, that the central object probability bias was effective.

### Semantic bias

A two-way, repeated measures ANOVA was run with semantic relation (SR and NR) and exposure duration (250 and 1,500 ms) as within-subjects factors. This revealed no main effects of semantic relation or exposure duration on RTs (*F*s < 1). However, we did observe an interaction [*F*(1, 19) = 4.44, *p* = .049, *η*_p_^2^ = .189]. Follow-up paired *t* tests showed a nonsignificant bias toward the SR object at 250 ms (*t* < 1; 835 vs. 842 ms for SR vs. NR), but a significant bias toward the NR object at 1,500 ms [*t*(19) = 2.10, *p* = .049, d_z_ = .47; 839 and 826 ms, respectively]. Accuracy analyses showed no main effect of semantic relationship [*F*(1, 19) = 1.28, *p* = .273, *η*_p_^2^ = .063; 93.9 % and 94.9 %, respectively] nor exposure duration [*F*(1, 19) = 1.60, *p* = .222, *η*_p_^2^ = .077; 94.0 % and 94.7 %, respectively], and no interaction (*F* < 1).

Taken together, Experiments 1–3 suggest that task-irrelevant semantic information has a relatively slow-acting effect on attention, not onsetting until ~750 ms (see the dashed line in Fig. 3 for a summary). Importantly, this contribution takes effect much later than those in studies that render semantic relationships task-relevant. Here, the objects themselves did little more than act as placeholders for the target and distractor items. Nonetheless, semantic relationships between the objects influenced attentional allocation. This suggests that there is a steady accrual of semantic information, even when it is irrelevant to the task, which then biases attention.

In addition, the initial semantic bias appears to be transient (see Fig. 3), and later in the time course SR objects are actually inhibited (i.e., slower RTs to targets that appear on the semantically related object). This biasing toward SR, followed by a bias away from that same location at later epochs, strongly resembles the microgenesis of the spatial allocation of attention. Namely, summoning attention to a spatial location with a peripheral cue speeds up processing at the cued location at short intervals following the cue (i.e., 300 ms or less), but slows down processing at the cued location following longer intervals. This slowing at the cued location is reminiscent of inhibition of return (IOR: Klein, 2000; Posner & Cohen, 1984; Tipper, Jordan, & Weaver, 1999), characterized by attention being repelled away from the cued location (which is marked as having been visited) in favor of other locations. Our results are consistent with this pattern, suggesting that the semantic properties of an object serve to constrain the spatial allocation of attention (i.e., at shorter object exposures, spatial locations bounded by a semantically related object enjoy an attentional benefit, whereas at longer object exposures, those same spatial locations exhibit an inhibition similar to IOR).

## Experiment 4

Experiments 1–3 demonstrated that semantic bias emerges around 750 ms and then persists in the form of biasing attention either toward the semantically related object (750 and 1,250 ms) or toward the semantically nonrelated objects, at later object exposure durations (1,500 and 2,000 ms). In the present experiment, the aim was to guard against two alternative interpretations that could be put forth to explain the observed pattern of results. First, the observed semantic biases could have been a byproduct of the particular duration pairings. Notably, the gaps between the durations in each group varied (i.e., 1,250 – 750 = 500 ms; 1,500 – 250 = 1,250 ms; 2,000 – 500 = 1,500 ms). Although this should not have affected attentional biasing at the first time point in each pair (250, 500, and 750 ms), it may have had an effect on the later pairings (1,250, 1,500, and 2,000 ms). As a control, in the next two experiments the object exposure times before the onsets of the target and distractors were decoupled. In Experiment 4, the object exposure durations (750 and 1,500 ms) were split between participants. If the initial SR bias is robust, it should be replicated. If the later NR bias is robust, it should also be replicated.

A second possibility is that, because targets were more likely to appear on the central object (50 %), this probability bias may somehow have tipped the scale toward the SR object. To control for this, in Experiment 4 the targets occurred on all three objects equally, reducing the relevance of the central object and, by extension, the SR object. Additionally, participants completed a posttest questionnaire on which they indicated whether they believed any factors predicted the target locations.

This experiment, therefore, served three purposes: it (i) provided an internal replication of the observed findings, (ii) decoupled any possible contributing effects of the object exposure pairings, and (iii) removed potential biases associated with the unequal probabilities of target presentation.

## Method

### Participants

The participants were assigned into one of two groups, with exposure durations of either 750 ms (31 participants: 19 female, mean age 20.0 years) or 1,500 ms (32 participants: 22 female, mean age 19.1 years) exposure duration. All were from GWU, gave informed consent, and were naïve to the purpose of the experiment. All experimental procedures were approved by the GWU Institutional Review Board.

### Stimuli and design

The stimuli and design were the same as in Experiments 1–3, with the following exceptions. Targets were equally likely to appear on any of the three objects, and the exposure duration was either 750 or 1,500 ms, varied between participants.

### Procedure

The experimental paradigm was the same as Experiment 1, except that participants were given 15 practice trials, followed by eight blocks of 80 trials. RT and accuracy feedback was given at the end of each block. The experiment lasted approximately 45–50 min.

## Results and discussion

In each group, one participant was removed for poor accuracy and one for having an overall median RT over 2.5 standard deviations from the grand mean (four removed, cumulatively). The median RTs are shown in Table 1. Unlike in Experiments 1–3, there was no probability manipulation, and thus we conducted a 3 × 2 mixed-design ANOVA with target location (central, SR, and NR) as the within-subjects factor and exposure duration as the between-subjects factor (750 and 1,500 ms). A main effect of target location emerged [*F*(2, 114) = 6.64, *p* = .002, *η*_p_^2^ = .104], with the fastest responses to targets on the central object and the slowest to those on the NR object (736, 758, and 764 ms for the central, SR, and NR objects, respectively), but no effect of exposure duration [*F*(1, 57) = 1.11, *p* = .296, *η*_p_^2^ = .019] and no interaction [*F*(2, 114) = 1.21, *p* = .302, *η*_p_^2^ = .021]. Paired *t* tests showed that a target on the central object still led to faster RTs than when it was on either the SR or the NR object (*t*s > 2.26, *p*s < .028). Critically, RTs were significantly faster to SR than to NR objects [*t*(58) = 2.52, *p* = .015, d_z_ = .33]. The accuracy analyses yielded no main effects or an interaction (*F*s < 1.67).

The results corroborate Experiment 1, with a significant bias toward the semantically related object. Thus, the semantic bias observed at 750 ms in Experiment 1 was indeed replicated. Interestingly, however, the 1,500 ms bias toward semantically nonrelated object was not replicated. This suggests that the previous NR bias was either a weak effect or could have resulted from a particular pairing of object exposures.

### Questionnaire

In the postexperiment questionnaire, we asked participants, in order: (1) Did you notice anything about the relationships between objects? If so, what do you think it was? (2) Did you notice anything about the frequency of where the target appeared? If so, what do you think it was? (3) If you were told that there was an imbalance in where the target appeared, what do you think it would be? Questions 1 and 2 were designed to motivate the participants to think about the objects they had just seen, and the final question was the one we used to gauge how many of the participants falsely assumed that targets appeared more often on the semantically related objects.

Only one participant in each experiment wrongly assumed that semantic information predicted the target location, and both guessed that the target occurred more often on the NR object (which did not bear out in their results). The fact that participants did not guess that semantic relationship predicted the target location strongly suggests that the semantic relationships were treated as task-irrelevant.

To summarize, we replicated Experiment 1’s results, with an attentional benefit for the semantically related object following a 750-ms object exposure duration. Given that the central object was less relevant in Experiment 4 (target 33 % of the time) than in Experiment 1 (target 50 % of the time), the reduced difference between the central, SR, and NR objects was to be expected. In addition to providing internal replication of the main finding, Experiment 4 eliminated two alternative interpretations: (i) that the semantic bias observed in the original experiment was simply due to the 50 % probability bias on the central object, and (ii) that participants were intentionally using semantic information to bias attentional allocation. The results support the hypothesis that objects’ semantic information biases the spatial allocation of attention, even when it is not predictive. Interestingly, the NR bias was not replicated, perhaps suggesting that either the previous finding was weak, in the nature of the effect, or was a result of the exposure duration pairs. Since this effect was not germane to the present investigation, we did not conduct any further follow-up studies.

## Experiment 5

The results of Experiments 1–4 suggest that there is an initial, transient bias to a semantically related object, even when semantic information is not predictive. However, we continued to find shorter RTs to the central object even when it was not favored by a probability bias. This suggests that participants were still attending to the central object first, and that the semantic properties of this object then influenced attentional allocation. This raises the question of whether an object needs to be attended for it to semantically influence attention. In Experiment 5, the targets never occurred on the central object but only on the SR and NR objects, 50 % of the time each. The central object was therefore always visible but would draw minimal attention, since the target would never appear there. If a visible object semantically biases attention even when it is minimally attended, we should still find a semantic bias. Conversely, if an object needs to be attended to exert a semantic-based attentional bias, we should fail to see a semantic bias when the central object never contains the target.

## Method

### Participants

Thirty-two participants took part (24 female, mean age 19.0 years). All were from GWU, gave informed consent, and were naïve to the purpose of the experiment. The experimental procedures were approved by the GWU Institutional Review Board.

### Stimuli, design, and procedure

The stimuli and design were the same as in Experiments 1–4, with the following exceptions. Targets never appeared on the central object, and appeared equally often (50 % of the time) on either the semantically related or the nonrelated object. The only exposure duration was 750 ms.

## Results and discussion

Two participants were removed, one for having below 80 % accuracy and one for having an overall median RT over 2.5 standard deviations from the grand mean. This left 30 participants’ data to analyze. A paired *t* test with semantic relation (SR and NR) as the factor revealed no difference in median RTs (*t* < 1, 677 and 680 ms, respectively) nor in accuracy [*t*(29) = 1.43, *p* = .164, d_z_ = .26; 95.0 % and 94.5 %, respectively]. This result suggests that for an object’s semantics to influence attentional allocation, that object (here, the central object) has to be attentionally selected first. The mere presence of an object is insufficient to produce a semantic bias on attentional allocation.

Taken together, Experiments 1–5 demonstrated that task-irrelevant semantic information biases attentional allocation. This is a relatively slow-acting, transient process, and requires that an object be spatially attended.

## Experiments 6, 7, and 8

In the next set of experiments, we examined the robustness of the semantic-biasing effect. In all of the previous experiments, the semantic relationship did not predict the target location, nor did any other factor (apart from the central-object probability bias in the first four experiments). The resulting semantic bias could therefore have been a result of the visual system “defaulting” to using semantic information as a guiding factor in the absence of other predictive factors. In real-world situations, inevitably some factor can be relied upon to help find a target (e.g., if looking for how much an item in a supermarket costs, you would be primed to look at the shelf underneath for the price tag). If semantic biasing robustly influences attentional allocation, it should persist even when a predictive factor is present.

Previous research has shown that semantic biasing of attention can coexist with other factors. De Groot et al. (2016) found in their search task that visual and semantic factors independently bias attention. Similarly, Belke et al. (2008) found that perceptual load did not affect the semantic biasing of attention, and that although cognitive load did affect semantically related objects, it did so only after an object had been selected. In neither case did these factors predict the target location. Experiments 6–8 probed the robustness of the semantic bias by including a predictive factor: a spatial probability bias. Instead of the targets being equally distributed among the SR and NR objects, now they were more likely (37.5 %) to appear on one side of the screen than the other (12.5 %; e.g., the left object would receive more targets than the right, independent of whether its object was or was not semantically related). Given this design, the high-probability location half of the time would coincide with the SR object, and half of the time with the NR object, again making semantic relatedness completely task-irrelevant. The peripheral side of the screen with the increased spatial probability was counterbalanced across participants. If semantic properties influence attentional allocation independently of other predictive factors, then we would continue to see an SR attentional bias. Conversely, if a strongly predictive factor (spatial probability) makes semantic information no longer necessary, then such information would no longer influence attentional allocation. Participants were assigned to 750–1,250 ms, 500–2,000 ms, and 250–1,500 ms exposure duration groups, allowing for a direct comparison with the results obtained from Experiments 1–3.

## Method

### Participants

Twenty-eight participants took part (24 female, mean age 19.9 years) in Experiment 6; 30 participants (22 female, mean age 19.4 years) in Experiment 7; and 26 participants (20 female, mean age 19.5 years) in Experiment 8. All were from GWU, gave informed consent, and were naïve to the purpose of the experiment. All experimental procedures were approved by the GWU Institutional Review Board.

### Stimuli and design

Experiment 6 mirrored the design of Experiment 1, with targets appearing 750 or 1,250 ms after peripheral object onset; in Experiment 7, targets appeared after 500 or 2,000 ms exposure durations, mirroring Experiment 2; and Experiment 8 had exposure durations of 250 and 1,500 ms, mirroring Experiment 3. Importantly, in all three experiments, the targets appeared on the central object 50 % of the time, and on the high- and low-probability sides of the screen 37.5 % and 12.5 % of the time, respectively. The high-probability side of the screen was counterbalanced across participants.

### Procedure

In all three experiments, the experiment began with 15 practice trials, followed by eight blocks of 80 trials. RT and accuracy feedback was given at the end of each block, and the exposure durations were randomized. Each experiment lasted approximately 45–50 min.

## Results

### Experiment 6

**Fig. 4:**
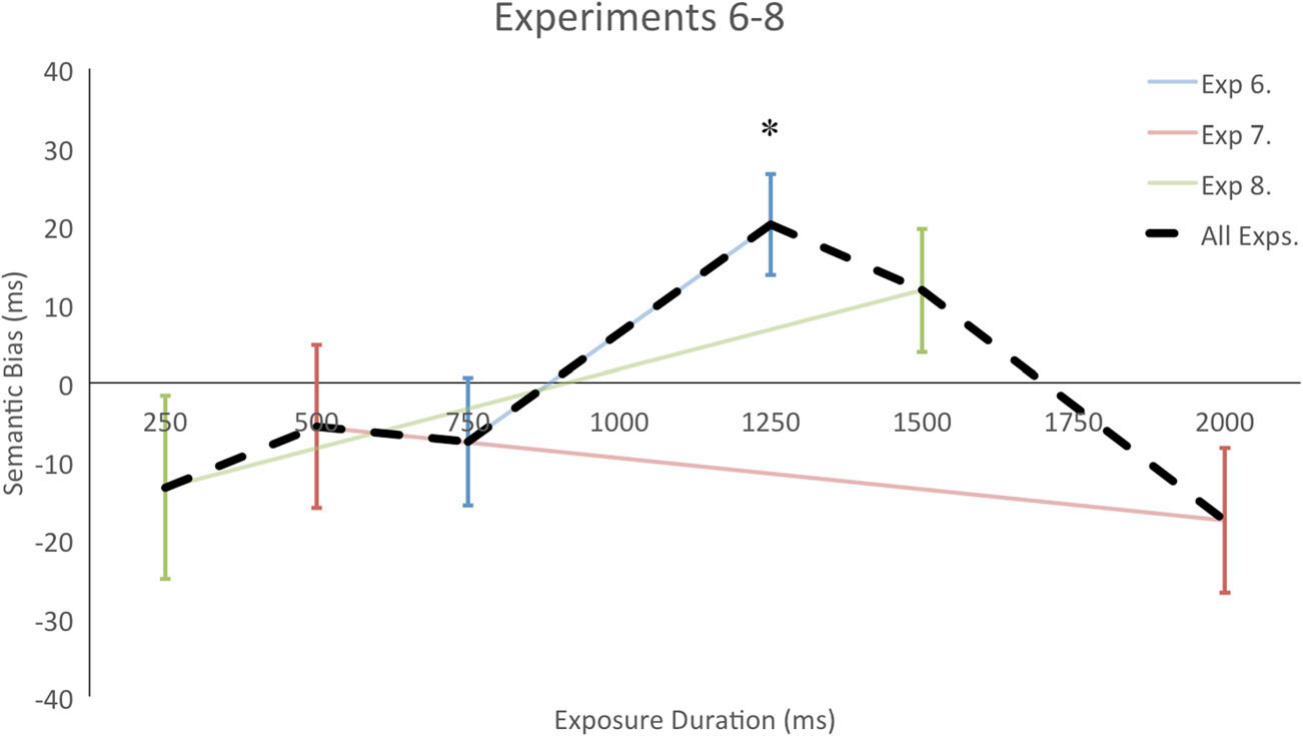
Experiments 6–8, which included a spatial probability bias toward the left or right side: Semantic biases (differences in response times between the nonrelated [NR] and semantically related [SR] conditions) over the six exposure durations. Positive numbers represent a bias toward the SR object; negative numbers, a bias toward the NR object. Error bars represent one standard error

The data were analyzed similarly to those from Experiments 1–5, except now the first block of experimental trials was removed, ensuring that we only analyzed trials after the spatial probability bias was learned. Two participants were removed for having accuracy less than 80 %. Response times are shown in Table 1 (see Fig. 4 for a visual summary).

We concentrated our analyses on the peripheral objects. A within-subjects 2 × 2 × 2 ANOVA was conducted, with semantic relationship (SR and NR), target location (peripheral high-probability and peripheral low-probability), and exposure duration (750 and 1,250 ms) as the factors. We observed a main effect of target location, with faster responses to the peripheral high-probability object [*F*(1, 25) = 19.61, *p* < .001, *η*_p_^2^ = .440; peripheral high-probability = 799 ms, peripheral low probability = 875 ms], and an interaction between semantic relationship and exposure duration [*F*(1, 25) = 6.25, *p* = .019, *η*_p_^2^ = .200]. The interaction was driven by the absence of a difference between SR and NR RTs at 750 ms (*t* < 1) and the presence of a significant bias toward the SR object at 1,250 ms [*t*(25) = 3.12, *p* = .005, d_z_ = .61; 825 vs. 845 ms for SR vs. NR, respectively]. None of the other effects were significant (*F*s < 1.78, *p*s > .195) in RTs, and accuracy analysis did not yield any significant main effects or interactions (*F*s < 1.40, *p*s > .248).

### Experiment 7

The data were analyzed similarly to those from Experiment 6. One participant was removed for having an accuracy less than 80 %, and one for having an overall median RT over 2.5 standard deviations over the grand mean. Response times are shown in Table 1.

A 2 × 2 × 2 repeated measure ANOVA was conducted on median RTs and accuracy, with semantic relationship (SR and NR), target location (peripheral high-probability and peripheral low-probability), and exposure duration (500 and 2,000 ms) as the factors. We found a main effect of target location, with shorter RTs to the peripheral high-probability location [*F*(1, 27) = 32.28, *p* < .001, *η*_p_^2^ = .544; 777 and 841 ms, respectively, for high and low probability], but no other analyses were significant (*F*s < 2.89, *p*s > .100). There was a strong trend toward a main effect of target location on accuracy [*F*(1, 27) = 3.97, *p* = .056, *η*_p_^2^ = .128], with higher accuracy in the peripheral high-probability condition (SR = 94.6 %, NR = 93.2 %), but no other main effect or significant interaction emerged for accuracy (*F*s < 2.74, *p*s > .110).

### Experiment 8

The data were analyzed similarly to those from Experiments 6 and 7. One participant was removed for having an overall median RT over 2.5 standard deviations from the grand mean. The removal criteria left an extra participant in the right-spatially-biased group, so the final participant tested in this particular group was therefore removed, in order to leave equal numbers of participants biased to the left and right. In all, there were 24 participants remaining for analysis. Response times are shown in Table 1.

A 2 × 2 × 2 repeated measure ANOVA was run on median RTs and accuracy, with semantic relationship (SR and NR), target location (peripheral high-probability and peripheral low-probability), and exposure duration (250 and 1,500 ms) as the factors. This revealed a main effect of target location, with shorter RTs to the peripheral high-probability location [*F*(1, 23) = 45.26, *p* < .001, *η*_p_^2^ = .663; 785 vs. 856 ms, respectively, for high vs. low probability], and a very weak trend toward an interaction between semantic relation and exposure duration [*F*(1, 23) = 3.08, *p* = .092, *η*_p_^2^ = .118]. No other analyses were significant (*F*s < 2.69, *p*s > .115). When we analyzed accuracy, a main effect of target location emerged, with higher accuracy at the peripheral high-probability location [*F*(1, 23) = 13.82, *p* = .001, *η*_p_^2^ = .375; 93.9 % and 91.0 %, respectively], but no other main effect or interaction was significant (*F*s < 2.31, *p*s > .143).

### Questionnaire

None of the participants in Experiments 6–8 indicated that they believed semantic relationships predicted the target location, indicating that high-level information was explicitly treated as task-irrelevant.

## Discussion

Experiments 6–8 were designed to test the robustness of the semantic bias when a more predictive factor was available. Despite participants indicating that they did not believe that semantic information was relevant, and the clear effect that the spatial probability bias had, object semantic information continued to bias attention. The results suggest that the visual system continually, in an online manner, processes the semantic information of attended objects and uses it to bias attention, rather than only “defaulting” to semantic information when no other guiding factor is available.

This finding goes beyond other studies that have indicated a continual influence of semantic relationships, by demonstrating that this influence is independent of other factors, such as visual similarities (de Groot et al., 2016) or perceptual load (Belke et al., 2008). In the present case the other factor, a spatial probability bias, predicted where the target would occur. This may explain why, whereas the previous studies had shown minimal changes to the semantic biasing of attention, we found a delayed onset, not peaking until 1,250 ms.

To verify whether this delayed onset of the semantic bias was statistically significant, we compared Experiments 1 and 6. Both experiments were based on exposure durations of 750 and 1,250 ms, and both showed a semantic bias of attention. However, in Experiment 1 this effect was stronger at 750, but in Experiment 6 it was only significant at 1,250 ms. We ran a 2 × 2 × 2 mixed-design ANOVA with experiment (balanced and spatial bias) as the between-subjects factor, and semantic relation (SR and NR) and exposure duration (750 and 1,250 ms) as the within-subjects factors.

We found a main effect of semantic relationship [*F*(1, 45) = 7.46, *p* = .009, *η*_p_^2^ = .142], with a bias toward the SR object (849 and 861 ms, respectively, for SR and NR) and a trend toward faster RTs in the 1,250 ms condition [*F*(1, 45) = 3.64, *p* = .069, *η*_p_^2^ = .071; 862 and 848 ms, respectively, for 750 and 1,250 ms]. Critically, we also observed a three-way interaction between experiment, semantic relation, and exposure duration [*F*(1, 45) = 7.88, *p* = .007, *η*_p_^2^ = .149]. Paired *t* tests showed that there was a semantic bias toward the SR object at 750 ms in the balanced experiment [*t*(20) = 4.32, *p* < .001], but not at 1,250 ms (*t* < 1); conversely, in the spatially biased experiment, there was no effect at 750 ms (*t* < 1), but a bias toward the SR object at 1,250 ms [*t*(25) = 3.12, *p* = .005]. No other main effect or interaction was significant (*F*s < 1.75, *p*s > .196). The results therefore suggest that the presence of a predictive cue delays, but does not extinguish, semantic biasing of attention.

## Image analysis: Color and size

Even though the earliest time point for observing semantic influences (750 ms) was rather late to be driven by low-level factors (i.e., color and size), we set out to formally rule out this possibility. To do so, we compared the differences in feature space between the central objects and their respective SR objects with the differences between the central objects and their respective NR objects. Individual objects were cut out and converted into LAB color space, which breaks down pixel information into a luminance channel and red–green and blue–yellow color channels. Histograms were made for each channel of each object, and bin-to-bin comparisons were run, summing the differences between the central object and its respective SR and NR objects. The smaller the difference between objects, the greater the similarity in that channel. If the results were due to low-level similarities, we should find significantly smaller differences in one or more channels between the central and SR objects than between the central and NR objects. Paired *t* tests revealed that none of the three channels had significant differences (*t*s < 1). The object sizes were then determined by pixel count, and a similar comparison between the central object and the SR and NR objects was again made, with smaller differences suggesting similar sizes. A paired *t* test failed to show a significant effect [*t*(19) = 1.02, *p* = .322, d_z_=.23]. These results suggest that the observed semantic bias was not a result of systematic low-level feature similarities.

## General discussion

When viewing real-world environments, humans prefer to attend to objects over empty backgrounds (Einhäuser, Rutishauser, & Koch, 2008; Hwang et al., 2011; Land, Mennie, & Rusted, 1999; Mack & Eckstein, 2011; Nuthmann & Henderson, 2010; Pajak & Nuthmann, 2013; Yarbus, 1967), making object properties an integral part of attentional biasing. Although the effects of low-level object properties such as boundaries and colors on attention have been well documented (e.g., Egly et al., 1994; Theeuwes, 1994; Treisman & Gelade, 1980; Wolfe, 1994), the effect of semantic information—a property inherently available in any recognizable object—is less well understood. Previous research focused on situations in which semantic information was relevant to an ongoing task (e.g., storing an item in visual working memory: Belke et al., 2008; de Groot, Huettig, & Olivers, 2015; Hwang et al., 2011; Mack & Eckstein, 2011; Moores et al., 2003; Telling, Meyer, & Humphreys, 2010). However, objects’ contextual relations are readily processed (Bar & Aminoff, 2003; Bar et al., 2008) and readily available, suggesting that semantic information could continually bias attentional allocation, even when it is task-irrelevant.

Over eight experiments, we demonstrated that nonpredictive semantic information biases attentional allocation. Experiments 1–3 showed an initial bias to the SR object, beginning around 750 ms after onset. This time is markedly later than what de Groot et al. (2016) found, but where they had made semantic information a critical component to the task (the meaning of the target was what separated it from the distractors), here semantic information was irrelevant, thus pushing the effect to occur later in time. This semantic bias was also found to be transient, and in fact reversed to favor the NR object at later time points (1,500 and 2,000 ms). Experiment 4 showed that this effect was not due to a target location bias toward the central object, since it still occurred even when targets were split evenly among the three objects. However, Experiment 5 showed that the central object at least has to be attended to as simply seeing the object was not enough to trigger a semantic bias of attention. These results strongly suggest that the visual system is sensitive to the semantic properties of attended objects—even when they do not predict the target location—which bias surrounding attention to semantically similar items.

In Experiments 6–8, we tested whether this semantic bias only constrains attentional allocation in the absence of other, more predictive cues. We found that attention was again biased to the SR object, although the onset of this effect was pushed further back, from 750 to 1,250 ms, but the late NR bias found at 1,500 and 2,000 ms in Experiments 2 and 3 were not replicated in Experiments 7 and 8. This could have been due to the NR effect being weak, or else to it being delayed, like the SR bias, and occurring at a longer time than we probed in these experiments. The delayed bias to the SR object contrasts with those from other studies, which showed that semantic biasing of attention was independent of other factors (visual similarity, perceptual load, etc.); however, in the present case the other factor was predictive of the target location. A potential explanation for the present results thus might be that in the presence of an apparent attentional strategy (in this case, based on probability), the influence of task-irrelevant semantic information is integrated into attentional guidance only if enough time has passed after the objects appeared and before targets are presented. Further research will have to be conducted to understand this effect. In summary, the results of Experiments 6–8 suggest that semantic information’s bias on attention is ongoing and robust.

The results reported here support the hypothesis that semantic biasing is not exclusive to task-relevant situations, but is an ongoing factor that continually affects attentional allocation. The combined results suggest that high-level semantic information complements low-level sensory properties in forming a bidirectional approach to allocating attention. It also opens the question of the mechanism through which these high-level factors may affect the attentional distribution. Previous research has suggested that an object’s contextual associations are derived in the parahippocampal and retrosplenial cortices (Bar & Aminoff, 2003; Livne & Bar, 2016). These representations could potentially affect the spatially organized attentional priority map in inferior parietal sulcus (IPS, Sheremata & Silver, 2015), either directly, or indirectly by influencing object recognition processing regions in the inferior temporal cortex (Bar, 2004), before influencing activity in IPS.

## Conclusion

The visual representation of the world biases our attention in consistent ways, independent of the viewer’s task. For example, the effect of low-level object properties (edges, color, etc.) on the spatial allocation of attention is well-established. However, the present results demonstrate that even higher-level properties such as semantic information affect attentional bias. High-level semantic properties therefore play an integral role in ongoing attentional biases within the visual system. If we are to develop a predictive and generalizable visual attention model in real-world settings, the relative semantic properties of objects will have to be incorporated.
